# High-Throughput Sequencing of Grapevine in Mexico Reveals a High Incidence of Viruses including a New Member of the Genus *Enamovirus*

**DOI:** 10.3390/v15071561

**Published:** 2023-07-16

**Authors:** Alfredo Diaz-Lara, Kristian Stevens, Vivian Hayde Aguilar-Molina, José Miguel Fernández-Cortés, Víctor Manuel Chabacano León, Marcos De Donato, Ashutosh Sharma, Teresa M. Erickson, Maher Al Rwahnih

**Affiliations:** 1 School of Engineering and Sciences, Tecnologico de Monterrey, Campus Queretaro, Queretaro 76130, Mexico; a01209069@tec.mx (V.H.A.-M.); a01208134@tec.mx (J.M.F.-C.); mdedonate@tec.mx (M.D.D.); asharma@tec.mx (A.S.); 2 Departments of Computer Science and Evolution and Ecology, University of California-Davis, Davis, CA 95616, USA; kastevens@ucdavis.edu; 3 Foundation Plant Services, University of California-Davis, Davis, CA 95616, USA; tmerickson@ucdavis.edu (T.M.E.); malrwahnih@ucdavis.edu (M.A.R.); 4 Facultad de Química, Universidad Autónoma de Querétaro, Santiago de Queretaro, Queretaro 76010, Mexico; victorchabacano@hotmail.com

**Keywords:** grapevine viruses, Mexico, diagnosis, sequencing, diversity, novel virus, real-time RT-PCR, epidemiology

## Abstract

This is the first viral metagenomic analysis of grapevine conducted in Mexico. During the summer of 2021, 48 plants displaying virus-like symptoms were sampled in Queretaro, an important grapevine-producing area of Mexico, and analyzed for the presence of viruses via high-throughput sequencing (HTS). The results of HTS were verified by real-time RT-PCR following a standardized testing scheme (Protocol 2010). Fourteen different viruses were identified, including grapevine asteroid mosaic-associated virus (GAMaV), grapevine Cabernet Sauvignon reovirus (GCSV), grapevine fanleaf virus (GFLV), grapevine fleck virus (GFkV), grapevine Pinot gris virus (GPGV), grapevine red globe virus (GRGV), grapevine rupestris stem pitting-associated virus (GRSPaV), grapevine rupestris vein feathering virus (GRVFV), grapevine Syrah virus 1 (GSyV-1), grapevine virus B (GVB), and grapevine leafroll-associated viruses 1, 2, 3, 4 (GLRaV1, 2, 3, 4). Additionally, divergent variants of GLRaV4 and GFkV, and a novel *Enamovirus*-like virus were discovered. This is the first report of GAMaV, GCSV, GLRaV4, GPGV, GRGV, GRVFV, and GSyV-1 infecting grapevines in Mexico; the impact of these pathogens on production is unknown.

## 1. Introduction

Grapevines (*Vitis* spp.) are one of the world’s most economically important horticultural crops, used for both food and wine production [1]. In Mexico, they are one of the most profitable crops, with 36,000 cultivated hectares producing 452,000 tons of grapes worth 11 billion Mexican pesos per year [2]. This plant hosts the most viral agents among perennial species, with more than 90 viruses that cause several diseases, some of which impact grapevine productivity [3,4,5]. Prior to this research, the only grapevine viruses partially documented in Mexico were grapevine fanleaf virus (GFLV), grapevine fleck virus (GFkV), grapevine red blotch virus (GRBV), grapevine rupestris stem pitting-associated virus (GRSPaV), grapevine leafroll-associated viruses 1, 2, 3 (GLRaV1, GLRaV2, GLRaV3), and grapevine viruses A and B (GVA, GVB); however, as discussed by [6], most reports are anecdotal sources lacking a confirmatory study. Little work has been done on the diagnostics and monitoring of grapevine viruses in Mexico, and there are no reports on their economic impact [7].

High-throughput sequencing (HTS) is a revolutionary molecular technique for plant virus diagnostics [8]. The technology allows the sequencing of a great number of nucleotides with high redundancy in a relatively short period of time. When combined with bioinformatics, it is a powerful tool for the detection of known and unknown viruses [9,10]. Compared to other single virus detection approaches, such as bioassays, enzyme-linked immunosorbent assay (ELISA), and various PCR formats, HTS enables us to study the complete virome of a plant species in a single assay [8]. As a result, HTS can be used to investigate the etiology of plant diseases, which has resulted in the discovery of several new viruses [8,9,11]. HTS does not require prior knowledge of viral genomic information or immunological characteristics that are not available for novel viruses.

In Mexico, there are limited studies on plant viruses using HTS. These studies include the detection of viruses in citrus trees, berries, beans, and non-cultivated plants [12,13,14,15,16]. HTS has not been applied to grapevines, despite the potential of the technique to know the phytosanitary status of this crop in the country [7]. For this reason, we surveyed grapevines in Queretaro, one of the most significant wine-producing entities in Mexico. Queretaro is the main producer and exporter of sparkling wine in Mexico with more than 3 million bottles produced annually (https://www.avq.com.mx/; accessed on 1 June 2023). The quality of this wine has been recognized internationally, as evidenced by multiple awards (medals) in international competitions (https://resultats.concoursmondial.com/fr/resultats/; accessed on 1 June 2023).

Grapevines displaying virus-like symptoms were collected from commercial vineyards and analyzed via HTS. A confirmatory test of putative viral sequences was provided by real-time RT-PCR following a standardized testing scheme. Findings include the first report of grapevine asteroid mosaic-associated virus (GAMaV), grapevine Cabernet Sauvignon reovirus (GCSV), grapevine leafroll-associated virus 4 (GLRaV4), grapevine Pinot gris virus (GPGV), grapevine red globe virus (GRGV), grapevine rupestris vein feathering virus (GRVFV), and grapevine Syrah virus 1 (GSyV-1) infecting grapevines in Mexico, and the discovery of divergent variants of GLRaV4 and GFkV, and a new *Enamovirus*-like virus.

## 2. Materials and Methods

### 2.1. Plant Material and RNA Extraction

**Figure 1:**
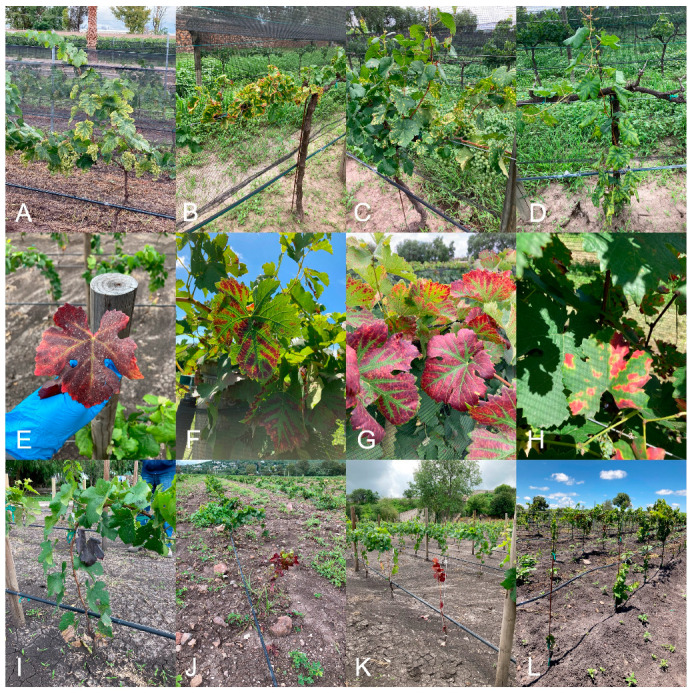
Virus-like symptoms on select grapevine plants that were sampled during the study. (**A**,**B**) plants showing chlorosis, yellowing and necrosis on leaves; (**C**) grapevine with leaf spotting; (**D**) plant displaying leaf roll; (**E**–**I**) plants showing reddening on leaves; (**J**–**L**) grapevines displaying dwarfism.

In the summer of 2021, 48 grapevine leaf samples were collected from 13 commercial vineyards located across the State of Queretaro, Mexico. The climate in Queretaro is semi-arid at an elevation of 1859 m above sea level and an average annual precipitation of 570 mm. At the time, although some plants were asymptomatic, others presented foliar and fruit necrosis, dwarfism, leaf spotting, curling, reddening, yellowing and chlorosis (Figure 1). Diverse white and red cultivars, such as Cabernet, Cabernet franc, Cabernet Sauvignon, Chardonnay, Gewürztraminer, Macabeo, Malbec, Merlot, Nebiolo, Parrellada, Pinot noir, Riesling, Syrah, Tempranillo, and Xarel-lo were sampled. Most of the plant material originated from France, Germany and Spain, and was imported to Mexico starting in 2010.

For sample preparation, 0.5 g of leaf petioles were cut from the grapevine samples and flash frozen in liquid nitrogen before grinding. Quick-RNA Plant Miniprep Kit (Zymo Research, Irvine, CA, USA) spin columns were used according to the manufacturer’s protocol. RNA concentration and quality was determined using Qubit (ThermoFisher Scientific, Sunnyvale, CA, USA) and Qsep100 (BiOptic Inc., New Taipei City, Taiwan), for which the required parameters were as follows: RQN > 8; minimum RNA amount per sample = 500 ng.

### 2.2. HTS and Virus Identification

Library construction of nucleic acids was carried out with the TruSeq Stranded Total RNA with Ribo-Zero Plant Kit (Illumina, San Diego, CA, USA), followed by the assembly of the obtained cDNA libraries after repairing their ends or converting them for ligation with the adapters by unique dual-indexes. Finally, sequencing was performed in an Illumina NovaSeq 6000 (Illumina, San Diego, CA, USA), with a flow cell SP Reagent Kit (2 × 100 cycles), located at the National Genomic Sequencing Laboratory Tec-BASE (Tecnologico de Monterrey, Monterrey, Mexico).

Illumina bcl2fastq2 software was used to perform demultiplexing and adapter trimming. Later, de novo assembly was performed using SPAdes software [17]. To annotate the de novo assemblies, contigs aligning to the grapevine genome at over 90% coverage using GMAP were removed [18]. The remaining contigs greater than 200 base pairs (bp) were compared to the GenBank non-redundant database of nucleotide sequences using BLASTn and BLASTx. Virus like sequences with significant hits to the known plant infecting virus families (*E*-value < 1 × 10^−5^) were further inspected to confirm their likely viral homology and host range based on their closest hits in GenBank.

### 2.3. Genome and Phylogenetic Analyses

The putative proteins and potential open reading frames (ORFs) encoded by virus like contigs determined by HTS were identified using ORFfinder and subsequent BLASTp annotation. To identify conserved domains within these proteins, the Pfam database [19] was searched using HMMER v3.1 [20]. Following sequence analysis, new virus sequences were submitted to GenBank.

Alignments of the sequences of viruses here identified and sequences from GenBank with the highest identity were carried out in MEGAX [21]. Briefly, genomic segments present in these viruses, including sequence of RNA-dependent RNA polymerase (RdRp) and coat protein (CP), were aligned by MUSCLE [22]. For the phylogenetic analysis, a Bayesian method was conducted using MrBayes v3.2.1 [23], implementing the general time-reversible (GTR) model with the rate at each site as random variable with a gamma distribution (G) and a proportion of invariable sites. Markov chain Monte Carlo (MCMC) chains were carried out for 10,000,000 generations.

### 2.4. Real-Time RT-PCR Validation of HTS

Following the viral metagenomic analysis, real-time RT-PCR assays were employed to confirm the presence of known viruses. Such assays are part of Protocol 2010, and they are used routinely at Foundation Plant Services (FPS, University of California-Davis, USA) as part of the grapevine certification program (https://fps.ucdavis.edu/fgr2010.cfm; accessed on 1 June 2023). These molecular tests have been described previously [24,25,26,27,28]. Some assays have been recently updated and are available from FPS by request.

Real-time RT-PCR reactions were completed in the QuantStudio 6 Flex Real-Time PCR System using the TaqMan Fast Virus 1-Step Master Mix (ThermoFisher Scientific, Sunnyvale, CA, USA) as per the manufacturer’s specifications. Each reaction (10 µL final volume) included 2 µL of RNA and final primer and probe concentrations of 900 and 250 nM, respectively. In addition, virus-specific assays were multiplexed with an 18S rRNA assay to confirm presence of RNA [24].

## 3. Results

### 3.1. HTS Data and Viral Sequences

Forty-eight grapevine plants originating from commercial vineyards located in Queretaro, Mexico were screened for viruses via HTS (Table S1). The paired end HTS protocol yielded between 15 and 67 million read pairs (220 bp in size) per cDNA library.

Metagenomic analysis identified sequences of several diverse viruses and viroids in the transcriptome of both symptomatic and asymptomatic sampled grapevines. Not a single plant was free of viral agents, though the composition of the infecting viruses was diverse. Of the total read counts, the majority of sequence belongs to the host plant; only 0.02–2% reads mapped to viruses or viroids.

### 3.2. Identification of Known Viral Agents Infecting Grapevine

Known viruses and viroids were identified in the analyzed plants as single or mixed infections (Table 1 and Table S2). Viruses included: GAMaV, GCSV, GFLV, GFkV, GPGV, GRGV, GRSPaV, GRVFV, GsyV-1, GVB, and GLRaV1, 2, 3, 4. For each distinct virus species, we selected the longest sequence (i.e., near complete genome) as representative of the virus; later, these sequences were annotated for their protein coding regions and deposited in GenBank. Viroids included grapevine hammerhead viroid-like RNA (GHVd) and grapevine yellow speckle viroids 1, 2, 3 (GYSVd1, 2, 3). GRSPaV was the most common viral agent, being detected in 46 out of 48 samples. On the other hand, GCSV, GFLV, GLRaV1, GLRaV2, GLRaV3, and GVB were identified infecting a single plant. In this survey, mixed infections predominated; one sample contained eight different viral agents (Vitis-TrPADL16).

### 3.3. New Genetic Diversity of Grapevine Viruses

The BLASTn annotation of the assembled contigs revealed a genetically diverse set of sequences from viruses known to infect grapevine. We identified the genomes of 14 viruses, represented by 116 distinct isolates (GRSPaV, 46 isolates; GRVFV, 18 isolates; GRGV, 19 isolates; GPGV, 10 isolates; GFLV, 1 isolate; GVB, 1 isolate; GFkV, 4 isolates; GsyV-1, 5 isolates; GCSV, 1 isolate; GAMaV, 4 isolates; GLRaV1, 1 isolate; GLRaV2, 1 isolate; GLRaV3, 1 isolate; GLRaV4, 4 isolates) among the 48 samples we analyzed. The nucleotide identity of our sequence to the closest virus homolog in GenBank is provided in Table 2.

As indicated in Table 2, this study provides substantial new diversity for six of the 14 grapevine infecting viruses. Further, if we consider 95% nucleotide identity as a tentative cutoff for a divergent isolate, we obtained a total of 36 new divergent isolates (GFkV, 4; GFLV, 1; GLRaV4, 4; GRGV, 6; GRSPaV, 8; GRVFV, 13). Interestingly, two well-studied and economically important grapevine viruses, GFkV and GLRaV4, displayed genetic diversity too, which was subsequently confirmed by a more robust phylogenetic analysis (Figures S1 and S2). The viral agent with the greatest amount of nucleotide diversity and largest number of divergent isolates was GRVFV.

### 3.4. Characterization of Novel Enamovirus-Like Virus

**Figure 2:**
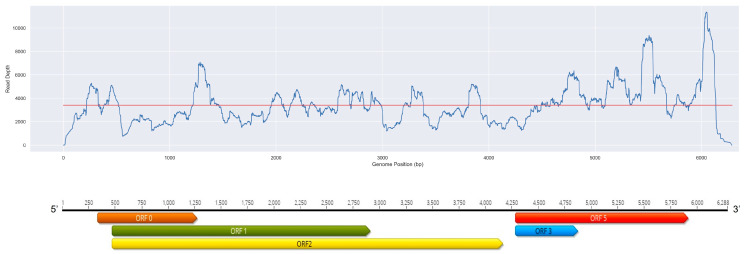
Genome organization of grapevine enamovirus 2 and plot of the read coverage over the genome. The organization and annotation of the open reading frames (ORFs) is typical of enamoviruses, ORFs indicated by different colors.

A separate annotation was performed to identify potential novel grapevine viruses in these samples, characterized by divergent protein homology to a virus known to infect plants. Consequently, a contig generated from the sample “Vitis-TrPADL13” showed a distant protein homology to grapevine enamovirus 1 (GEV1, genus *Enamovirus*, family *Solemoviridae*). The new viral sequence displayed an average depth of 3411 reads (Figure 2).

**Figure 3:**
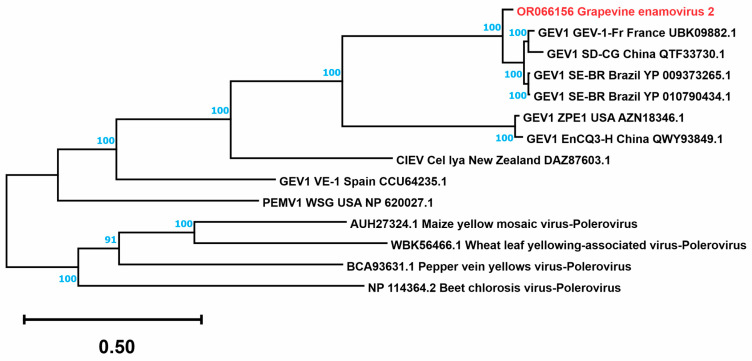
Tree resulting from the Bayesian phylogenetic analysis using the DNA sequence of the coat + read through protein (CP-RTD) of the enamoviruses and using poleroviruses as outgroup. Sequences were obtained from the GenBank; accession numbers are shown. The percentages of replicate trees in which the associated taxa clustered together in the bootstrap test (10,000,000 iterations) are shown next to the branches. The tree is drawn to scale with branch lengths in the same units as those of the evolutionary distances used to infer the phylogenetic tree, which are the number of base substitutions per site. The tree with the highest log likelihood (−11,550.44) is shown. The sequence identified in this study is shown in red.

The near complete genome of the putative novel virus, named grapevine enamovirus 2 (GEV2), was determined to be 6288 nucleotide (nt) long (GenBank: OR066156). BLASTn comparisons revealed the closest homologous sequence as GEV1 isolate CS-BR with 87.91% nt identity (98% query coverage). The GEV2 genome (Figure 2) contains five ORFs organized like other enamoviruses. ORF 0 encodes a protein of 313 amino acids (aa) (34.45 kDa), which was similar (76% aa identity, 99% query coverage) to the P0 protein GEV1. ORF 1 is 814 codons in length and its translational product (89.99 kDa) is most related to the P1 protein of GEV1 (85% aa identity, 100% query coverage) and contains a peptidase S39 super family (K_294_-S_490_). ORF 2-encoded protein (1232 aa, 136.88 kDa) has the highest similarity to the GEV1 RdRp (88% aa identity, 100% query coverage), and it is produced by ribosomal slippage. A CP with a molecular weight of 21.86 kDa is encoded by ORF 3 (197 aa), based on 85% aa identity (100% query coverage) with the corresponding product of GEV1; additionally, a luteo coat super family (F_62_-N_195_) motif was also identified. Lastly, the ORF 5 545 aa-long protein (readthrough protein) is related to an aphid transmission protein of GEV1 (60.24 kDa) and shares 90% aa identity (100% query coverage) with the ortholog protein expressed by the GEV2 genome. Finally, phylogenetic analysis confirmed the relation of GEV2 with other enamoviruses, including GEV1 (Figure 3).

### 3.5. Detection of Known Grapevine Viruses by Real-Time RT-PCR

Real-time RT-PCR was used to confirm the presence of grapevine viruses identified by HTS. Virus detection was then validated by comparing HTS and real-time RT-PCR results for each sample. The real-time RT-PCR assays produced Ct values that ranged from 20 to 39 (Table S3).

## 4. Discussion

In this study, we completed the first viral metagenomic analysis of grapevine in Mexico. HTS results were validated using a standardized virus detection protocol, which involves real-time RT-PCR assays. A set of 48 grapevine plants, the majority displaying virus-like symptoms, was found to be infected by 14 viruses and 5 viroids; additionally, a new potential member of the genus *Enamovirus* was discovered. The high incidence of viral agents calls for an improvement in the sanitation protocols and management of grapevine viruses in Mexico. Previously, we discussed the risk posed by viral pathogens for grapevine production in this country [7]. Some of the viruses here identified are vectored by arthropods and nematodes, in addition to virus transmission via plant propagation material, consequently, this may contribute to the spread of viral diseases in Mexican vineyards.

Grapevine plants analyzed during this study were collected from different commercial vineyards along the State of Queretaro. Overall, the average age of these vineyards was 8 years old. Mexico lacks nurseries and propagation programs with large-scale operations; thus, most of the stock planting material originated from abroad. The European origin of some of these plants may explain the presence of viruses linked to serious economic losses in the old world, like GFLV and GPGV [29,30,31]. On the other hand, GRBV, an economically important pathogen in the USA, and previously reported in Baja California, Mexico was not detected during this survey [32,33].

We compared our virus sequences to reference genomes available in public databases to determine genetic diversity. The most divergent viruses were GFkV, GLRaV4, GRGV, and GRVFV. In the case of GFkV and GLRaV4, phylogenetic analysis suggests that these novel variants do not belong to any previously known type (i.e., group or cluster), and instead represent potentially new subclades. Thus, the near-complete genome sequences for these viruses create the first base-line framework for major grapevine viruses and their genetic variants identified in Mexico.

In 2017, GEV1 was described infecting grapevine in Brazil, being the first report of a virus in the genus *Enamovirus* infecting this host [34]. Criteria used to demarcate species of this genus include differences in aa sequence identity of any gene product greater than 10% (https://ictv.global/report/chapter/solemoviridae/solemoviridae/enamovirus; accessed on 1 May 2023). Consequently, based on sequence identity, the putative novel virus found in sample “Vitis-TrPADL13” represents a new species belonging to the genus *Enamovirus*, which we provisionally name GEV2. Given the presence of additional viral agents in this sample, it was not possible to ascertain whether GEV2 is associated with symptoms. Complementary experiments on its pathogenicity are needed, likewise, studies investigating the distribution of this new virus.

This work establishes the first report of virus and viroid populations infecting grapevine in Queretaro. Similar field surveys should be performed in other important grapevine growing regions in Mexico, such as Sonora, Zacatecas and Coahuila. By contrast, initial efforts have already been done in Aguascalientes and Baja California through small-scale surveys [32,35]. Epidemiological studies aiming to prevent the introduction and movement of virus-infected grape material within Mexico.

According to our results, mixed infections were common among the analyzed samples, being GRSPaV frequently detected. Initially, GRSPaV was considered the causal agent of Rupestris stem pitting, a common graft-transmitted disease. However, subsequent studies have shown that GRSPaV does not affect growth or develop symptoms in cultivars such as Albano, Madeleine Sylvaner, Ortega, and Savagnin rose [36]. In addition, the same asymptomatic grapevines did not show yield reduction or other chemical changes that would affect berry quality [37]. The virus has also been associated with Syrah decline, vein necrosis, or other vein affectation on Chardonnay, but this has not been fully corroborated. Therefore, GRSPaV is mostly considered a latent virus that has evolved to coexist with its host [38]. Lastly, this viral agent may be transmitted through pollen and seeds, vegetative propagation, and grafting [39,40].

Although the grapevine industry is relatively small in Mexico, some of the oldest vineyards in the American continent are situated in the country, being important sources of genetic diversity that must be preserved. As a result, the first search for grapevine viruses via HTS was conducted in Mexico. To our knowledge, this is the initial report of GAMaV, GCSV, GLRaV4, GPGV, GRGV, GRVFV, and GSyV-1 infecting grapevines in Mexico; in all cases, detection was confirmed by two independent analyses (i.e., HTS and RT-PCR). To prevent future negative impacts on the local grapevine industry, new plantations should prioritize the use of certified clean stock, with virus diagnosis and tissue culture as requirements, to provide the means for long-term elimination of viral diseases from vineyards.

## Figures and Tables

**Table 1:** Viral agents identified in grapevine plants via high-throughput sequencing (HTS). Plants originated from commercial vineyards located in Queretaro, Mexico.

Sample ID	GLRaV1	GLRaV2	GLRaV3	GLRaV4	GFLV	GPGV	GFkV	GAMaV	GRGV	GRSPaV	GRVFV	GsyV-1	GVB	GCSV	GYSVd1	GYSVd2	GYSVd3	GHVd
Vitis-TrPADL1										+					+			
Vitis-TrPADL2									+	+	+				+			
Vitis-TrPADL3									+	+	+				+			
Vitis-TrPADL4										+	+				+			
Vitis-TrPADL5									+	+	+				+			
Vitis-TrPADL6						+				+					+			
Vitis-TrPADL7										+	+				+			
Vitis-TrPADL8									+	+	+				+			
Vitis-TrPADL9									+	+								
Vitis-TrPADL10										+		+			+			
Vitis-TrPADL11								+	+	+	+				+			
Vitis-TrPADL12										+					+	+		
Vitis-TrPADL13									+	+						+		
Vitis-TrPADL14				+	+					+	+				+			
Vitis-TrPADL15										+	+				+	+		
Vitis-TrPADL16			+			+	+		+	+		+	+		+			
Vitis-TrPADL17								+		+					+			
Vitis-TrPADL18									+	+	+				+			
Vitis-TrPADL19									+	+								
Vitis-TrPADL20				+						+					+			
Vitis-TrPADL21										+	+				+			
Vitis-TrPADL22									+	+		+			+			
Vitis-TrPADL23									+	+	+				+			
Vitis-TrPADL24		+								+					+			
Vitis-TrPADL25										+					+			
Vitis-TrPADL26								+	+	+	+				+			
Vitis-TrPADL27	+									+					+			
Vitis-TrPADL28										+				+	+			
Vitis-TrPADL29						+				+					+			
Vitis-TrPADL30						+				+					+			+
Vitis-TrPADL31										+					+			
Vitis-TrPADL32						+			+	+					+			
Vitis-TrPADL33						+		+	+	+					+			
Vitis-TrPADL34										+		+					+	
Vitis-TrPADL35									+	+	+							
Vitis-TrPADL36															+			+
Vitis-TrPADL37						+				+					+			
Vitis-TrPADL38						+				+					+			
Vitis-TrPADL39															+			
Vitis-TrPADL40										+					+			
Vitis-TrPADL41										+					+			
Vitis-TrPADL42				+					+	+	+				+		+	
Vitis-TrPADL43							+			+					+			
Vitis-TrPADL44									+	+	+				+			
Vitis-TrPADL45							+			+	+	+			+			
Vitis-TrPADL46				+		+	+		+	+	+				+			
Vitis-TrPADL47						+				+					+			
Vitis-TrPADL48										+								

Grapevine rupestris stem pitting-associated virus (GRSPaV), grapevine rupestris vein feathering virus (GRVFV), grapevine red globe virus (GRGV), grapevine Pinot gris virus (GPGV), grapevine fanleaf virus (GFLV), grapevine virus B (GVB), grapevine fleck virus (GFkV), grapevine Syrah virus-1 (GsyV-1), grapevine Cabernet Sauvignon reovirus (GCSV), grapevine asteroid mosaic-associated virus (GAMaV), grapevine leafroll-associated viruses 1, 2, 3, 4 (GLRaV1, 2, 3, 4), grapevine hammerhead viroid-like RNA (GHVd), and grapevine yellow speckle viroids 1, 2, 3 (GYSVd1, 2, 3).

**Table 2:** New divergent isolates of grapevine viruses and their closest homolog in GenBank.

Sample ID	Contig Length	Virus	% ID	Top Hit ID *
Vitis-TrPADL16	4538	GFkV	84	MN716779.1
Vitis-TrPADL45	4614	GFkV	85	MN716779.1
Vitis-TrPADL43	2550	GFkV	91	MN716779.1
Vitis-TrPADL46	2149	GFkV	95	MN716779.1
Vitis-TrPADL14	7294	GFLV	88	MH492668.1
Vitis-TrPADL46	1506	GLRaV4	91	JX513893.1
Vitis-TrPADL20	13,814	GLRaV4	94	JX513893.1
Vitis-TrPADL42	3839	GLRaV4	95	JX513893.1
Vitis-TrPADL14	7582	GLRaV4	95	JX513893.1
Vitis-TrPADL33	1762	GRGV	88	MZ451072.1
Vitis-TrPADL42	1819	GRGV	91	KX109927.1
Vitis-TrPADL35	2894	GRGV	91	MZ451067.1
Vitis-TrPADL46	3863	GRGV	92	NC_030693.1
Vitis-TrPADL47	2583	GRGV	93	MZ451070.1
Vitis-TrPADL44	1478	GRGV	94	MZ451069.1
Vitis-TrPADL42	6289	GRSPaV	88	MG938298.1
Vitis-TrPADL17	7613	GRSPaV	88	MG938310.1
Vitis-TrPADL14	8710	GRSPaV	88	MG938298.1
Vitis-TrPADL9	4266	GRSPaV	93	MN228487.1
Vitis-TrPADL37	8629	GRSPaV	93	KX274277.1
Vitis-TrPADL13	8704	GRSPaV	93	KX274277.1
Vitis-TrPADL48	8779	GRSPaV	93	KX274277.1
Vitis-TrPADL47	9034	GRSPaV	93	AY368172.2
Vitis-TrPADL26	2861	GRVFV	83	MZ027155.1
Vitis-TrPADL46	6854	GRVFV	84	MT084811.1
Vitis-TrPADL5	1763	GRVFV	85	MZ027155.1
Vitis-TrPADL27	2823	GRVFV	85	MZ451100.1
Vitis-TrPADL23	3821	GRVFV	85	MT084814.1
Vitis-TrPADL35	3986	GRVFV	86	MZ451083.1
Vitis-TrPADL17	4152	GRVFV	86	MZ027155.1
Vitis-TrPADL11	5988	GRVFV	86	MZ027155.1
Vitis-TrPADL45	6729	GRVFV	87	MN974276.1
Vitis-TrPADL43	6772	GRVFV	87	MZ027155.1
Vitis-TrPADL42	6853	GRVFV	87	MZ027155.1
Vitis-TrPADL15	2241	GRVFV	88	MZ451078.1
Vitis-TrPADL14	3252	GRVFV	88	MZ027155.1

Grapevine rupestris stem pitting-associated virus (GRSPaV), grapevine rupestris vein feathering virus (GRVFV), grapevine red globe virus (GRGV), grapevine fanleaf virus (GFLV), grapevine fleck virus (GFkV), grapevine leafroll-associated virus 4 (GLRaV4). Identity for GenBank accession number (*). High blue color intensity denotes higher nucleotide diversity.

## Data Availability

All sequencing data obtained in this study were included in the manuscript and/or submitted to the GenBank database under the accession numbers OR066156 and BioProject PRJNA989637.

## Supplementary Materials

The following supporting information can be downloaded at: https://www.mdpi.com/article/10.3390/v15071561/s1, Figure S1: Tree resulting from the Bayesian phylogenetic analysis using the DNA sequence of the coat protein of grapevine fleck virus; Figure S2: Tree resulting from the Bayesian phylogenetic analysis using the DNA sequence of the heat shock protein 70 of grapevine leafroll-associated virus 4; Table S1: Grapevine samples used for high-throughput sequencing; Table S2: Known viruses and viroids identified by high-throughput sequencing; Table S3: Detection of grapevine viruses by real-time RT-PCR assays.
