# Supplementary material for: High-Throughput Sequencing of Grapevine in Mexico Reveals a High Incidence of Viruses including a New Member of the Genus Enamovirus
Source: Viruses. 2023 Jul 16;15(7):1561. doi: 10.3390/v15071561 (PMC10386000; doi:10.3390/v15071561)
Supplement: Supplementary file 1 [file viruses-15-01561-s001.zip › Figure S2.pptx]

## Slide 1
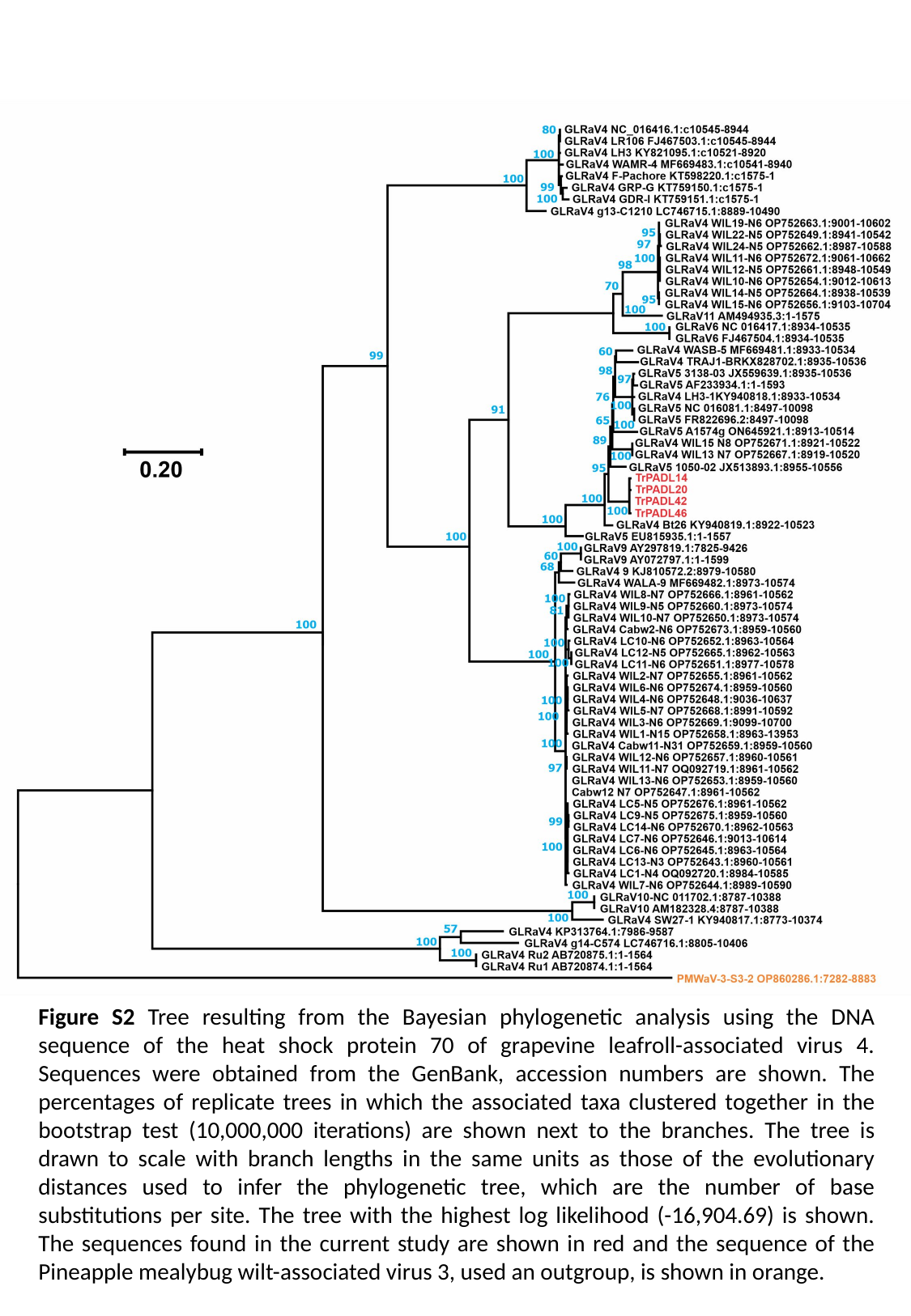

Figure S2 Tree resulting from the Bayesian phylogenetic analysis using the DNA sequence of the heat shock protein 70 of grapevine leafroll-associated virus 4. Sequences were obtained from the GenBank, accession numbers are shown. The percentages of replicate trees in which the associated taxa clustered together in the bootstrap test (10,000,000 iterations) are shown next to the branches. The tree is drawn to scale with branch lengths in the same units as those of the evolutionary distances used to infer the phylogenetic tree, which are the number of base substitutions per site. The tree with the highest log likelihood (-16,904.69) is shown. The sequences found in the current study are shown in red and the sequence of the Pineapple mealybug wilt-associated virus 3, used an outgroup, is shown in orange.
